# A Remarkable Case of Reflex Nervous Action

**Published:** 1852-11

**Authors:** J. W. Bright

**Affiliations:** Louisville, Ky.


Art. II.—*A Remarkable Case of Reflex Nervous Action.* By J. W. Bright, M. D., Louisville, Ky.

That nervous irritation in one part of the human system, by whatever cause it may have been excited, may be transferred to another part, or organ, is an occurrence so common that every practising physician is familiar with it. For instance, the irritation of a calculus in the bladder will give rise to a pain in the glans penis, or pains in the thighs. Now those sensations are not produced by a direct communication of sensation through the nervous cords, but the physical changes excited by the first, are reflected in the second. Again, when the stomach is affected by the action of a strong acid upon its coats, there is often a severe pain felt in the supra-orbital nerve. An antacid taken into the stomach quickly relieves this pain. A lump of ice swallowed suddenly will often produce a similar effect in the supra orbital nerves, and a draft of water warmer than the ice will as soon relieve it. My object, however, in this short notice, is to give an account of a remarkable case of reflex nervous action which I attended a short time since.

I was called, September 8th, 1852, to see Mrs. B——, about six months and a half advanced in her first pregnancy, a very delicate lady. She had been attacked the day before with a chill, followed by fever of the usual intermitting form, which was, by the use of the ordinary remedies, removed in two or three days. About twenty-four hours after all the symptoms of fever had been removed, pains set up in the uterus, simulating those of labor. I saw her in five or six hours after these pains commenced, her friends supposing that she was in labor. Her pains came on regularly, and were of considerable strength. On examination of the os uteri I found the pain was of spasmodic character, and not genuine labor pains—with nothing of the expulsive character attending such pains. An anodyne was ordered, (morphine and tinct. valerian,) which soon suspended them in the uterus. But in a very short time the spasmodic action was transferred to the diaphragm, and hiccup set in, which became very troublesome, and resisted all the usual remedies for twenty-four hours, till finally it yielded to a preparation of creosote, sulph. æther and laudanum.

But notwithstanding she was under the influence of the above medicine, as soon as the hiccup began to subside the pain returned in the uterus, and by the time the hiccup was entirely relieved, the uterine pains were as severe as at first. I allowed them to continue for eight or ten hours, but no evidences of delivery appeared. The os uteri remained firm, and the head of the child made no descent. I then suspended the uterine pains once more by the use of McMunn's elixir of opium. Sixty drops had the effect, and she immediately commenced hiccuping again; but after a few spasmodic actions of the diaphragm the action passed to the brain, or membranes of the brain, and she fell into a profound stupor. In this state her pulse was one hundred and five in a minute, soft and compressible; her breathing only nine times in a minute. She could be aroused by shaking and talking loud to her, but would instantly fall into a deep stupor again. The pupil of the eye at this time was very much contracted, and not sensible to light. There was frequent knitting of the eyebrows. She could talk and swallow when aroused, but the pulse was too soft to bear the lancet. I ordered an active cathartic, to be followed by an enema. This operated well, but no relief was obtained. I then ordered strong cataplasms of mustard to be applied over the stomach and extremities, for they were too cool, and gave a full dose of calomel, and again met it by an enema, which also acted well. No relief to the brain. I then resolved to excite the uterus, and as soon as I effected it, and the pain was set up there, and labor commenced, she began to arouse and talk, and look about the room. In six hours from the time the uterus was again in active operation the child was born, and she was fully awake, and perfectly conscious of every thing that was passing.

Thus, it will be remarked, forty-eight hours passed before the uterus was made to act, and her brain relieved.

The child lived about twenty-four hours. The mother recovered without any difficulty. The remarkable character of this case has induced me to give it to the profession. The treatment is nothing more than any experienced member of the profession would have adopted under such circumstances. It is the reflex action of the nervous system shown in this case to which I wish to direct the attention of the profession. I leave it without comment. Although I have practised medicine for nearly forty years, I never saw exactly such a case before. I am induced to believe that they are rare, and, when they do occur, we need not expect relief until delivery is accomplished.

LOUISVILLE, October, 1852.

